# Damselflies (Zygoptera: Odonata) of Pakistan: Part 1

**DOI:** 10.1673/031.011.10201

**Published:** 2011-08-16

**Authors:** Ahmed Zia, Muhammad Naeem, Muhammad Ather Rafi, Falak Naz, Sumera Afsheen, Muhammad Ilyas

**Affiliations:** ^1^ National Insect Museum, National Agriculture Research Centre, Islamabad — Pakistan; ^2^ Department of Entomology, PMAS Arid Agriculture University, Rawalpindi — Pakistan; ^3^ Department of Zoology, University of Gujrat — Pakistan

**Keywords:** *Elattoneura atkinson*, *Elattoneura souteri*, faunistics, *Libellago lineata lineata*, new records

## Abstract

The present study is an effort to document bio-geographical distribution for Zygoptera of Pakistan. Damselflies were collected throughout the country and territory of Azad Jammu and Kashmir during 2004–2009. A total of 2692 specimens were collected yielding 9 families, 21 genera, and 48 species and subspecies. Three of these species, *Libellago lineata lineata* (Burmeister), *Elattoneura atkinsoni* (Selys), and *Elattoneura souteri* (Fraser), are recorded for the first time from Pakistan. Distribution, habitats, previous records, and Zoogeographic affiliation for all collected taxa are discussed. Help was also taken from published literature on Zygoptera of Pakistan, and specimens housed at National Insect Museum were also studied. In total, 53 species are accounted for providing an updated record for all modern taxa of damselfly fauna of Pakistan.

## Introduction

Pakistan is situated between latitudes of 23° 35' to 37° 05′ North and longitudes of 60° 50′ to 77° 50′ East. It stretches over 1,600 km north to south and 885 km east to west, with a total area of 796,096 km^2^. The country has a sub-tropical and semi-arid climate with annual rainfall ranges from 125 mm in extreme southern plains to 500–900 mm in the submountainous and northern plains. About 70% of total rainfall occurs as heavy downpours in summer during July to September and 30% in winter. Summer except in mountains is very hot, with an average maximum temperature of 40° C while the minimum temperature in the winter is a few degrees above the freezing point (Atlas of Pakistan 1997).

Most scientists are aware of difficulties faced during faunistic studies in a country like Pakistan, where different corners of the country overlap with different regions of the world. On a biogeographical basis, a major part of Pakistan is Palearctic (Hindu Kush, Karakorum, western Himalayas, Sulaiman Range, North Pakistan sandy desert, and western Indus Valley), while the rest of the area is Oriental (Indus River Delta, Eastern Indus Valley Desert, Thar desert, Rann of Kutch in southern Punjab, and eastern Himalayas) with traces of Ethiopian or Afrotropical regions (southern Iran to extreme southwestern of Baluchistan). Hindu Kush, Karakorum, and the Himalayas are major biogeographic boundaries between subtropical and tropical flora and fauna of Indian subcontinent and temperate climate Palearctic ecozone (Rafi et al. 2010).

Pakistan has abundance of Oriental, Palearctic, and Ethiopian (Afrotropical) fauna. It is interesting to point out that insect fauna confirm the transitional position of Pakistan. Here the Oriental representation of species is continuous with those of Indian Punjab and Rajisthan, and Palearctic is continuous with those of Iranian Baluchistan, eastern Afghanistan, and Russia (separated by only a few miles) and northwestern and eastern China. Also, there is an Ethiopian influence which runs along the southern coastal areas of Sindh and eastern Mekran in Baluchistan (Qadri 1968).

Odonates have been a focus of extensive research in many countries. They are one of the few insect orders that have been intensively studied in the tropics (Woodward 2001). They have been reported from all continents except Antarctica, and are usually concentrated in warmer and tropical habitats (Boyd 2005). According to Trueman and Rowe (2001), approximately 6500 named species of Odonata have been described so far all over the world. In comparison, Pakistan stands far behind even from its ecologically similar nearby countries (India 500 species, Srilanka 120 species, and Nepal with 180 species).

The distribution of damselflies is not well explored in Pakistan. Many scattered, but limited, studies have been carried out in the past (Appendix 1). Geopolitically, the country is in an important region of the world, with variable habitats and unlimited resources of water in the form of snow, streams, springs, and rivers. The objective of the present study is to explore Zygopterous fauna of Pakistan by doing extensive collection throughout the country.

## Materials and Methods

Surveys were carried out during summer season of six consecutive years (2004–2009) to collect adult damselflies from 171 localities in different districts of Pakistan. Surveyed areas include all four provinces (Punjab, Sindh, North West Frontier Province (N.W.F.P.), Baluchistan), the Northern Areas (NA), and Azad Jammu and Kashmir (AJ&K). Details of collection sites are given in Table 1.

Adult damselflies were caught with a light insect net during 1100 to 1900 on hot sunny days of spring and summer. The net had a 2ft long handle and a ring of about 25 cm in diameter with an open-mesh net. Damselflies were killed in glass jars containing potassium cyanide. After killing, specimens were placed in triangle envelopes with their wings folded over the body. Data regarding locality, date of collection, and collector's name were written on outside of envelope. Information about habitat was noted in a field book. In general, only one specimen was kept in each envelope so as to avoid damaging the specimens. Pairs caught during mating were placed in the same envelope. After being brought to the laboratory, specimens were placed in a humid chamber to soften them for spreading. As the specimens became soft they were shifted to moisture absorbent papers for a few minutes, pinned, and spread over wooden setting boards. After drying the specimens were labeled and moved to storage boxes. Naphthalene balls were mounted in storage boxes and anti-ant powder was sprinkled around the boxes within cabinets to prevent collection from attack of insects. Specimens were then identified up to species level by running them through keys following Fraser (1933–1934), Khaliq (1990), and Subramanian (2005). Voucher specimens were deposited in department of entomology PMAS Arid Agriculture University, Rawalpindi and their representatives were sent to National Insect Museum, NARC - Islamabad.

**Table 1.**
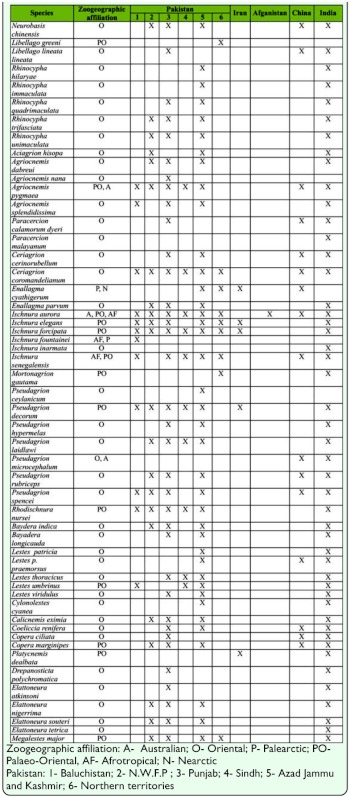
Presence of Zygoptera in Pakistan and its bordered neighboring countries.

## Results

A total of 2692 specimens were collected, yielding 9 families, 21 genera, and 48 species and subspecies. Among these, three species, *Libellago lineata lineata* (Burmeister), *Elattoneura atkinsoni* (Selys), and *Elattoneura souteri* (Fraser) are recorded for the first time from Pakistan. Details regarding valid names, distribution, habitat description, previous records from Pakistan, world distribution, Zoogeographic affiliation, and number of individual males and females collected are provided for all the species. As a whole, 53 species are accounted. Details for these species are provided below.

**Family Calopterygidae Selys, 1850 *Neurobasis chinensis* Linnaeus, 1758**
**Own Records:** Loc. 2 - 1 ♂*,* 2 ♀; Loc. 6 - 2 ♂, 2 ♀; Loc. 7 - 7 ♂, 5 ♀; Loc. 16 - 1 ♂, 1 ♀; Loc. 22 - 2 ♂, 1 ♀; Loc. 20 - 1 ♂; Loc. 37 2 ♂, 5 ♀; Loc. 38 - 1 ♂; Loc. 67 - 2 ♂, 3 ♀; Loc. 54 - 3 ♂, 5 ♀; Loc. 55 - 2 ♂; Loc. 53 2 ♂, 2 ♀; Loc. 127 - 1 ♂, 2 ♀; Loc. 113 - 1 ♂; Loc. 117 - 1 ♂, 1 ♀; Loc. 118 - 3 ♀; Loc. 119 - 4 ♂; Loc. 142 - 1 ♂.
**Previous Records from Pakistan:** Kanth (1985) reported this species for the first time from Azad Jammu and Kashmir, Pakistan. In 1999, Khaliq and Maula collected it from N.W.F.P., Pakistan. Recently Khan et al. (2008), Zia et al. (2008), and Rafi et al. (2009) reported it again from Azad Jammu and Kashmir.
**Notes:** Specimens were collected while flying over fast running water streams, sitting on submerged grasses, swampy places along banks of rivers, and from rice fields.
**World Distribution:** Throughout India, except desert (Fraser 1934); Hong Kong (Wilson 1997); Srilanka, India, Nepal, Bangladesh, Hong Kong, and South China (Ades and Kendrick 2004); China (Wilson et al. 2008); Singapore (Rashid et al. 2008).
**Zoogeographic Affiliation:** Oriental.

**Family Chlorocyphidae Cowley, 1937 *Libellago greeni* Laidlaw 1924**
**Own Records:** Loc. 147 - 2 ♂.
**Previous Records from Pakistan:** Zia et al. (2009) reported it from northern Pakistan (Gilgit; Danyore), which comes under Palearctic areas of Pakistan.
**Notes:** Specimens were collected while flying among dense vegetation along a moving water stream.
**World Distribution:** Srilanka (Fraser 1934; Silsby 2001; Bedjanic and Conniff pers. comm.).
**Zoogeographic Affiliation:** Palaeo-Oriental.

***Libellago lineata lineata* Burmeister, 1839**
**Own Records:** Loc. 2 - 6 ♂, 4 ♀.
**Previous Records from Pakistan:** Reported for the first time from Pakistan.
**Notes:** They were observed to be an active fliers. The genera *Elattoneura* and *Rhinocypha* were also collected from this area also. They were recorded from fast running and splashing water surrounded by bushes, scrubs, and grasses. Sometimes they sat over high grasses and tall vegetation, and at times moved within grasses and shrubs.
**World Distribution:** Sundaic Archipelago and North India (Fraser 1934); Vietnam (Vantol and Rozendaal 1995); India (Subramanian 2005, 2009); China (Wilson et al. 2008).
**Zoogeographic Affiliation:** Oriental.

***Rhinocypha hilaryae* Fraser, 1927**
**Own Records:** Loc. 128 - 1 ♂, 2 ♀.
**Previous Records from Pakistan:** Reported from Azad Jammu and Kashmir by Khaliq et al. (1990), Rafi et al. (2009), and Zia et al. (2008).
**Notes:** This species was found on vegetation surrounding out-flow of a water lake.
**World Distribution:** Upper Burma (Fraser 1934); India (Subramanian 2009).
**Zoogeographic Affiliation:** Oriental.

***Rhinocypha immaculata* Selys, 1871**
**Own Records:** Loc. 126 - 2 ♂*,* 1 ♀; Loc. 127 - 2 ♀; Loc. 123 - 1 ♂*;* Loc. 132 - 1 ♀; Loc. 134 - 2 ♂; Loc. 117 - 1 ♂*,* 1 ♀; Loc. 118 - 1 ♀; Loc. 142 - 1 ♂; Loc. 143 - 1 ♀.
**Previous Records from Pakistan:** Previously reported from Azad Jammu and Kashmir by Khaliq et al. (1990), Zia et al. (2008), and Rafi et al. (2009).
**Notes:** The species was collected over slowmoving water with a lot of vegetation around it. The collection spot was a natural pasture for grazing animals, surrounded by thick forest plantations.
**World Distribution:** India (Fraser 1934; Subramanian 2009).
**Zoogeographic Affiliation:** Oriental.

***Rhinocypha quadrimaculata* Selys, 1853**
**Own Records:** Loc. 2 - 8 ♂, 3 9; Loc. 5 - 2 ♂, 1 ♀; Loc. 3 - 2 ♂, 2 ♀; Loc. 9 - 5 ♂*,* 3 ♀; Loc. 11 - 3 ♂; Loc. 14 - 2 ♂; Loc. 17 - 1 ♂, 2 ♀; Loc. 16 - 2 ♂, 3 ♀.
**Previous Records from Pakistan:** Khaliq (1990) reported this species from Punjab province. Kanth (1985), Khan et al. (2008), Zia et al. (2008), and Rafi et al. (2009) collected it from Azad Jammu and Kashmir.
**Notes:** Recorded from mountainous areas. Specimens were collected from the banks of seasonal water streams, and while sitting over big rock stones in perennial water bodies and rivers. Sometimes they were found perching over long grass stems in the water. A few specimens were found hiding between big gaps of stones at the edges of streams.
**World Distribution:** India (Fraser 1934; Subramanian 2009).
**Zoogeographic Affiliation:** Oriental.

***Rhinocypha trifasciata* Selys, 1853**
**Own Records:** Loc. 9 - 2 ♂; Loc. 17 - 1 ♂, 1 ♀; Loc. 18 - 3 ♂, 2 9; Loc. 68 - 1 ♂, 3 ♀; Loc. 77 - 1 ♂, 1 ♀; Loc. 58 - 4 ♂, 2 ♀; Loc. 133 - 1 ♂, 2 ♀; Loc. 128 - 1 ♂; Loc. 122 - 2 9; Loc. 121 - 2 ♂, 1 ♀; Loc. 124 - 1 ♂, 1 ♀; Loc. 135 - 2 ♂; Loc. 129 - 3 ♂, 5 ♀.
**Previous Records from Pakistan:** Khaliq (1990) documented this species from the N.W.F.P. and Punjab provinces. Khaliq and Maula (1999) also reported it from N.W.F.P. Khaliq et al. (1990), Zia et al. (2008), and Rafi et al. (2009) collected its specimens from Azad Jammu and Kashmir.
**Notes:** Collection was made from poorly vegetated banks of slow running water streams and from small bushes near streams. Some specimens were caught while sitting over peaks of half submerged small rocks.
**World Distribution:** India (Fraser 1934; Subramanian 2009).
**Zoogeographic Affiliation:** Oriental.

***Rhinocypha unimaculata* Selys, 1853**
**Own Records:** Loc. 5 - 4 ♂, 2 ♀; Loc. 6 - 4 ♂, 2 ♀; Loc. 11 - 1 ♂, 1 ♀; Loc. 17 - 3 ♂, 2 ♀; Loc. 13 - 1 ♂, 2 ♀; Loc. 14 - 1 ♂, 2 ♀; Loc. 76 - 1 ♂; Loc. 123 - 1 ♂, 2 ♀; Loc. 126 - 3 ♂, 2 ♀; Loc. 127 - 1 ♂, 1 ♀; Loc. 134 - 2 ♂, 1 ♀; Loc. 113 - 1 ♀; Loc. 117 - 1 ♀; Loc. 119 - 1 ♂; Loc. 142 - 1 ♂; Loc. 143 - 2 ♀.
**Previous Records from Pakistan:** Khaliq (1990) documented this species from the N.W.F.P. and Punjab provinces. Khaliq and Maula (1999) also reported it from N.W.F.P. Khan et al. (2008), Zia et al. (2008), and Rafi et al. (2009) collected specimens from Azad Jammu and Kashmir.
**Notes:** Specimens were collected while they were sitting over small submerged rocks and from rock stones around water streams. Mostly they were recorded from streams having no flora on their banks.
**World Distribution:** India (Fraser 1934; Subramanian 2009).
**Zoogeographic Affiliation:** Oriental.

**Family Coenagrionidae Kirby, 1890 *Aciagrion hisopa* Selys, 1876**
**Own Records:** Loc. 74 - 1 ♂, 2 ♀; Loc. 125 - 2 ♂, 3 ♀.
**Previous Records from Pakistan:** Kanth (1985) and Khaliq et al. (1990) recorded this species from Azad Jammu and Kashmir. Khaliq and Maula (1999) collected its specimens from N.W.F.P. Again Zia et al. (2008) and Rafi et al. (2009) collected this species from Azad Jammu and Kashmir.
**Notes:** Specimens were collected from running water spots and standing water pits with a lot of thin, grassy vegetation surrounding these spots.
**World Distribution:** Burma, Srilanka (Ceylon), Malaysia, India, Pulo Besoar, and Malay states (Fraser 1933); India (Subramanian 2005, 2009); Singapore (Rashid et al. 2008); Srilanka (Bedjanic and Conniff pers. comm.).
**Zoogeographic Affiliation:** Oriental.

***Agriocnemis dabreui* Fraser, 1919**
**Own Records:** Loc. 20 - 6 ♂, 2 ♀; Loc. 29 3 ♂, 1 ♀; Loc. 23 - 1 ♂, 1 ♀; Loc. 28 - 1 ♂; Loc. 24 - 3 ♂, 1 ♀; Loc. 19 - 3 ♂, 1 ♀; Loc. 51 - 1 ♂; Loc. 132 - 2 ♂, 1 ♀.
**Previous Records from Pakistan:** Khaliq (1990) reported this species from N.W.F.P. However, Zia et al. (2008) collected it from Azad Jammu and Kashmir.
**Notes:** Specimens were recorded from thin, as well as thick, grassy vegetation grown at banks of water streams. They were also collected from weedy stagnant water ponds.
**World Distribution:** India (Fraser 1933; Subramanian 2009).
**Zoogeographic Affiliation:** Oriental.

***Agriocnemis nana* Laidlaw, 1914**
**Own Records:** Loc. 40 - 2 ♂.
**Previous Records from Pakistan:** Khaliq (1990) reported this species from Punjab.
**Notes:** Specimens were collected from the bank of a river that had grasses growing along its margins.
**World Distribution:** Burma (Fraser 1933); Singapore (Rashid et al. 2008); Malaysia (Choong et al. 2008).
**Zoogeographic Affiliation:** Oriental.

***Agriocnemis pygmaea* Rambur, 1842**
**Own Records:** Loc. 28 - 3 ♂, 3 ♀; Loc. 23 2 ♂, 1♀; Loc. 20 - 5 ♂, 5 ♀; Loc. 21 - 9 ♂, 3 ♀; Loc. 29 - 5 ♂, 6 ♀; Loc. 22 - 4 ♂, 4 ♀; Loc. 33 - 4 ♂, 1 ♀; Loc. 34 - 6 ♂, 2 ♀; Loc. 31 - 1 ♂, 1 ♀; Loc. 35 - 3 ♂, 2 ♀; Loc. 32 - 2 ♂, 1 ♀; Loc. 2 - 5 ♂, 4 ♀; Loc. 4 - 2 ♂, 3 ♀; Loc. 7 - 1 ♂, 1 ♀; Loc. 19-2 ♂, 3 ♀; Loc. 43 - 3 ♂, 2 ♀; Loc. 42 - 5 ♂, 1 ♀; Loc. 45 - 2 ♂; Loc. 46 - 2 ♂; Loc. 47 - 2 ♂, 1 ♀; Loc. 48 - 1 ♂; Loc. 76 - 1 ♂, 4 ♀; Loc. 69 - 1 ♂, 2 ♀; Loc. 67 - 1 ♂; Loc. 76 - 2 ♀; Loc. 61 - 1 ♂; Loc. 59 1 ♂, 1 ♀; Loc. 63 - 1 & 3 ♀; Loc. 60 - 1 ♂, 1 ♀; Loc. 64 - 3 ♂, 1 ♀; Loc. 58 - 2 ♂; Loc. 52 2 ♂, 1 ♀; Loc. 56 - 1 ♂, 1 ♀; Loc. 49 - 5 ♂, 3 ♀; Loc. 57 - 4 ♂, 2 ♀; Loc. 80 - 2 & 1 ♀; Loc. 85 - 2 ♂, 1 ♀; Loc. 86 - 1 ♀; Loc. 89 - 3 ♀♂, 2 ♀; Loc. 88 - 3 ♂, 1 ♀; Loc. 95 - 2 ♂; Loc. 93 2 ♀; Loc. 90 - 1 ♂; Loc. 87 - 2 ♀; Loc. 91 - 2 ♂, 1 ♀; Loc. 90 - 1 ♀; Loc. 88 - 1 ♂; Loc. 106 - 1 ♂; Loc. 101 - 3 ♂, 2 ♀; Loc. 105 - 2 ♂, 1 ♀; Loc. 104 - 4 ♂, 1 ♀; Loc. 110 - 2 ♂, 2 ♀; Loc. 109 - 4 ♂; Loc. 98 - 2 ♂, 3 ♀; Loc. 102 2 ♂, 2 ♀; Loc. 100 - 2 ♂, 3 ♀; Loc. 107 - 1 ♀; Loc. 108 - 1 ♂; Loc. 123 - 3 ♂, 2 ♀; Loc. 129 1 ♂, 3 ♀; Loc. 133 - 2 ♂, 2 ♀; Loc. 131 - 4 ♂ 3 ♀; Loc. 132 - 1 ♂; Loc. 138 - 2 ♂; Loc. 140 5 ♂, 3 ♀; Loc. 112 - 2 ♂ 3 ♀; Loc. 116 - 1 & Loc. 127 - 6 ♂, 1 ♀; Loc. 120 - 3 ♂, 1 ♀; Loc. 145 - 1 ♂.
**Previous Records from Pakistan:** Kanth (1985) and Khaliq et al. (1990) reported this species from Azad Jammu and Kashmir. Khaliq (1990) documented it from all four provinces. Khaliq and Maula (1999) again collected this species from N.W.F.P. Khaliq and Siddique (1995), Zia et al. (2008), and Rafi et al. (2009) recorded it from Azad Jammu and Kashmir. Mitra and Babu (2009) reported its subspecies, *Agriocnemis p. pygmaea,* from Punjab province.
**Notes:** Collected from thick and dense vegetation grown along and a little away from water streams. Also collected from rice fields and marshy spots surrounded by heavy grassy vegetation.
**World Distribution:** Throughout the Oriental region, Australia, Pacific Islands, India, Burma, Singapore, Srilanka (Ceylon), Sydney, Formosa, China, N. Celebes, New Guinea, Philippines, Queensland, Seychelles, Java (Indonesia), Nicobars, and Manila (Fraser 1933); Hong Kong, Seychelles, Middle East, India, China, Japan, Indonesia, and Australia (Ades and Kendrick 2004); India (Subramanian 2005; 2009); China (Wilson et al. 2008); Singapore (Rashid et al. 2008); Srilanka (Bedjanic and Conniff pers. comm.).
**Zoogeographic Affiliation:** Paleo-oriental and Australian.

***Agriocnemis splendidissima* Laidlaw, 1919**
**Own Records:** Loc. 4 - 1 ♂; Loc. 19 - 2 ♂; Loc. 106 - 2 ♂, 1 ♀; Loc. 140 - 3 ♂, 2 ♀.
**Previous Records from Pakistan:** Khaliq (1990) reported this species from Baluchistan. Khaliq and Siddique (1995) and Zia et al. (2008) collected its specimens from Azad Jammu and Kashmir.
**Notes:** Specimens were found flying along running water, marshy areas, and feeding on aphids in rice fields.
**World Distribution:** India (Fraser 1933; Subramanian 2005, 2009).
**Zoogeographic Affiliation:** Oriental.

***Paracercion calamorum dyeri* Ris, 1916**
**Own Records:** Loc. 11 - 1 ♂, 1 ♀.
**Previous Records from Pakistan:** This subspecies was earlier reported from the Punjab province by Khaliq (1990) and Mitra and Babu (2009).
**Notes:** Specimens were collected from vegetation grown along the stagnant water of a pond and a small dam.
**World Distribution:** Hong Kong (Wilson 1997); India, Nepal, China, Japan, Hong Kong and Indonesia (Ades and Kendrick 2004); India (Subramanian 2005).
**Zoogeographic Affiliation:** Oriental.

***Paracercion malayanum* Selys, 1876**
**Own Records:** Nil.
**Previous Records from Pakistan:** Reported from Punjab province by Mitra and Babu (2009).
**World Distribution:** India (Subramanian 2009).
**Zoogeographic Affiliation:** As per reported distribution, it may be an Oriental species.

***Ceriagrion cerinorubellum* Brauer, 1865**
**Own Records:** Loc. 21 - 1 ♂; Loc. 22 - 1 ♂; Loc. 126 - 1 ♀; Loc. 112 - 2 ♂, 3 ♀; Loc. 114 - 2 ♂; Loc. 115 - 1 ♂.
**Previous Records from Pakistan:** Kanth (1985) collected this species from Azad Jammu and Kashmir. Khaliq (1990) reported it from the Punjab province. Khan et al. (2008), Zia et al. (2008), and Rafi et al. (2009) collected specimens from Azad Jammu and Kashmir.
**Notes:** Specimens were observed to be a submountanious species and they were found on dwarf vegetation grown near very slowmoving water channels as well as from tall vegetation present among and aside bogs and marshes.
**World Distribution:** Srilanka/Ceylon, India, Malaysia, Indonesia, and Borneo (Fraser 1933); Singapore (Rashid et al. 2008); Malaysia (Dow and Reels 2008); China (Choong et al. 2008); India (Subramanian 2005, 2009); Srilanka (Bedjanic and Conniff pers. comm.).
**Zoogeographic Affiliation:** Oriental.

***Ceriagrion coromandelianum* Fabricius, 1798**
**Own Records:** Loc. 21 - 2 ♂, 3 ♀; Loc. 27 1 ♂; Loc. 29 - 5 ♂, 4 ♀; Loc. 10 - 4 ♂, 2 ♀; Loc. 11 - 2 ♂, 1 ♀; Loc. 12 - 3 ♂, 3 ♀; Loc. 14 - 4 ♂, 4 ♀; Loc. 2 - 6 ♂, 5 ♀; Loc. 19 - 2 ♂, 1 ♀; Loc. 39 - 4 ♂, 2 ♀; Loc. 43 - 1 ♂, 2 ♀; Loc. 42 - 3 ♂, 2 ♀; Loc. 40 - 1 ♂, 3 ♀; Loc. 31 - 1 ♂, 1 ♀; Loc. 67 - 1 ♂, 1 ♀; Loc. 76 - 2 ♂, 3 ♀; Loc. 76 - 3 ♂, 3 ♀; Loc. 70 - 2 ♂, 1 ♀; Loc. 71 - 1 ♂; Loc. 52 - 5 ♂, 2 ♀; Loc. 78 - 1 ♂, 2 ♀; Loc. 87 - 1 ♂, 2 ♀; Loc. 110 - 1 ♂, 1 ♀; Loc. 110 - 3 ♂, 4 ♀; Loc. 111 - 1 ♂; Loc. 131 - 4 ♂, 2 ♀; Loc. 134 - 3 ♂, 4 ♀; Loc. 132 - 4 ♂, 6 ♀; Loc. 126 - 2 ♂, 5 ♀; Loc. 127 - 3 ♂, 1 ♀; Loc. 125 - 1 ♂; Loc. 113 - 1 ♂; Loc. 117-1 ♂, 1 ♀; Loc. 118 - 3 ♀; Loc. 142 - 1 ♂; Loc. 144 - 3 ♂, 1 ♀.
**Previous Records from Pakistan:** Kanth (1985) and Khaliq et al. (1990) collected specimens from Azad Jammu and Kashmir. Khaliq (1990) reported this species from Punjab, N.W.F.P., Sindh, and Baluchistan provinces. Khaliq and Maula (1999) also collected it from N.W.F.P. Zia et al. (2008) and Rafi et al. (2009) again documented it from Azad Jammu and Kashmir. Zia et al. (2009) reported its presence in northern Pakistan. Mitra and Babu (2009) reported it from the Sindh and Punjab provinces.
**Notes:** Specimens were collected from a variable number of ecological habitats, including rice fields, grasses grown near stagnant water, dwarf vegetation along water streams and lakes, and from weeds present on banks of very slow-running water ways. It was recorded from stagnant water ponds to perennial water flows of the country. Specimens were found in plains as well as on high peaks of mountains.
**World Distribution:** Throughout Srilanka/Ceylon, India, Malaysia, South China, and Indo-China (Fraser 1933); India (Subramanian 2005, 2009); Srilanka (Bedjanic and Conniff pers. comm.).
**Zoogeographic Affiliation:** Oriental.

***Enallagma cyathigerum* Charpantier, 1840**
**Own Records:** Loc. 167 - 3 ♂, 1 ♀; Loc. 169 - 3 ♀; Loc. 168 - 1 ♂, 2 ♀; Loc. 171 - 1 ♂, 4 ♀; Loc. 162 - 4 ♂, 4 ♀; Loc. 161 - 1 ♂, 1 ♀; Loc. 164 - 4 ♀; Loc. 163 - 5 ♂, 2 ♀; Loc. 166 - 6 ♂, 2 ♀; Loc. 160 - 6 ♂; Loc. 147 - 7 ♂, 4 ♀.
**Previous Records from Pakistan:** Khaliq et al. (1994) and Zia et al. (2009) reported specimens from northern Pakistan. Mitra and Babu (2009) reported the subspecies, *Enallagma c. cyathigerum,* from the Punjab province.
**Notes:** Specimens were recorded from grasses grown along standing water, lakes, and slowmoving water streams. Specimens were also collected from bushes and spiky plants present far away from Borith Lake at Hunzanagar (northern Pakistan). The majority of the collection spots were getting water due to the melting of snow on the mountains during the summer.
**World Distribution:** Central Asia including Kashmir and Tibet, Europe, North America, and British Isles (Fraser 1933); Europe, West Asia, Caucasia, and Middle East (Askew 1988); Spain (Jodicke 1994); Europe, Asia, and North America (Steimann 1997); Levant and Syria (Schneider 2004); Turkey (Kalkman et al. 2003; Salur and Ozsarac 2004; Salur and Mesci 2007; Miroglu and Kartal 2008); Iran (Ebrahimi et al. 2009).
**Zoogeographic Affiliation:** Palearctic and Nearctic.

***Enallagma parvum* Selys, 1876**
**Own Records:** Loc. 39 - 1 ♂; Loc. 50 - 1 ♂.
**Previous Records from Pakistan:** Kanth (1985) collected its specimens from Azad Jammu and Kashmir. Khaliq (1990) reported this species from the Punjab and N.W.F.P. provinces. Mitra and Babu (2009) reported it from the Sindh and Punjab provinces.
**Notes:** Specimens were collected from bogs and marshes.
**World Distribution:** South Asia, India, Burma, and Srilanka/Ceylon (Fraser 1933); India (Subramanian 2009); Srilanka (Bedjanic and Conniff pers. comm.).
**Zoogeographic Affiliation:** Oriental.

***Ischnura aurora* Brauer, 1865**
**Own Records:** Loc. 22 - 1 ♂; Loc. 29 - 3 ♀; Loc. 25 - 4 ♂, 2 ♀; Loc. 26 - 1 ♂, 1 ♀; Loc. 32 - 1 ♂, 1 ♀; Loc. 31 - 1 ♂; Loc. 11 - 2 ♂, 3 ♀; Loc. 14 - 2 ♀; Loc. 8 - 1 ♂, 2 ♀; Loc. 19 - 1 ♂, 3 ♀; Loc. 67 - 2 ♂, 3 ♀; Loc. 60 - 1 ♂; Loc. 64 - 4 ♂, 1 ♀; Loc. 59 - 1 ♂; Loc. 97 3 ♂; Loc. 132 - 1 ♂; Loc. 126 - 3 ♀; Loc. 125 - 1 ♂; Loc. 127 - 4 ♂, 1 ♀; Loc. 123 - 4 ♂, 3 ♀; Loc. 112 - 2 ♂, 3 ♀; Loc. 114 - 2 ♂; Loc. 113 - 1 ♂; Loc. 117 - 1 ♂, 1 ♀; Loc. 119 - 4 ♂; Loc. 142 - 1 ♂; Loc. 143 - 1 ♀; Loc. 140 - 1♂.
**Previous Records from Pakistan:** Kanth (1985) and Khaliq et al. (1990) reported this species from Azad Jammu and Kashmir. Khaliq (1990) documented its presence in Punjab, N.W.F.P., Sindh, and Baluchistan provinces. Khaliq and Maula (1999) also reported it from N.W.F.P. Zia et al. (2008) and Rafi et al. (2009) again collected it from Azad Jammu and Kashmir. Zia et al. (2009) reported it from northern Pakistan. Mitra and Babu (2009) reported the subspecies, *Ischnura a. aurora,* from Sindh and Punjab provinces.
**Notes:** Specimens were collected from thin grasses, swampy places, river banks, rice fields, marshes, and weedy water ponds. Sometimes specimens were found flying among submerged vegetation present along river banks. Being a very light-weight and small-sized species, air currents sometimes carry it far away and thus at times specimens were collected from unusual habitats.
**World Distribution:** South Asia, India, Srilanka (Ceylon), Burma, Malaysia, Sondaic Archipelago, Borneo, New Guinea, Australasia, Philippines, and Samoa (Fraser 1933); Afghanistan (Kimmins 1950); India (Subramanian 2005, 2009); Bhutan (Mitra 2006); Fiji (Evenhuis and Polhemus 2007); China (Wilson et al. 2008; Yu 2008). Nishida (2008) reported it from Moorea and indicated presence of this species in Africa, India, Srilanka, Australasian, Afrotropical, Indomalayan, and East Indies; Srilanka (Bedjanic and Conniff pers. comm.).
**Zoogeographic Affiliation:** Australian, Paleo-oriental, and Afrotropical.

***Ischnura elegans* Vander Linden, 1820**
**Own Records:** Loc. 22 - 1 ♂; Loc. 27 - 1 ♂; Loc. 30 - 1 ♂, 3 ♀; Loc. 7 - 1 ♂, 2 ♀; Loc. 12 - 2 ♂, 1 ♀; Loc. 76 - 1 ♂, 1 ♀; Loc. 126 3 ♀; Loc. 127 - 1 ♂, 2 ♀; Loc. 125 - 2 ♂; Loc. 165 - 1 ♂; Loc. 162 - 3 ♀; Loc. 161 - 1 ♂, 2 ♀; Loc. 164 - 2 ♂ 2 ♀; Loc. 163 - 3 ♂, 1 ♀; Loc. 166 - 4 ♂, 2 ♀; Loc. 169 - 1 ♂, 2 ♀; Loc. 157 - 1 ♂, 2 ♀; Loc. 158 - 2 ♀; Loc. 150 - 1 ♂, 1 ♀; Loc. 154 - 2 ♂, 2 ♀; Loc. 152 - 2 ♂, 1 ♀; Loc. 153 - 1 ♂, 4 ♀.
**Previous Records from Pakistan:** This species was reported for the first time by Fraser (1933) from N.W.F.P. and Baluchistan provinces. Kanth (1985) and Khaliq et al. (1990) reported the species from Azad Jamu and Kashmir. Khaliq (1990) documented its presence in the N.W.F.P. and Baluchistan provinces. Again Khaliq and Maula (1999) reported it from N.W.F.P. Khan et al. (2008), Zia et al. (2008), and Rafi et al. (2009) collected it from Azad Jammu and Kashmir. Zia et al. (2009) reported it from northern Pakistan. Mitra and Babu (2009) reported the subspecies, *Ischnura e. elegans,* from the N.W.F.P and Punjab provinces.
**Notes:** Collections were made from thin and grassy vegetation, rice fields, and marshy places near river banks. Sometimes specimens were found between submerged vegetation grown at river banks.
**World Distribution:** British Isles to Europe and mid-Asia, N.W.F.P., Baluchistan, and Seistan (Fraser 1933); South and Central Anatolia, the Levant, Turkey, and Crete (Dumont 1977; Steinmann 1997); all of Europe (except Iceland), south and middle Anatolia, Middle East (Askew 1988; Dumont 1991); Spain (Jodicke 1994); Russia (Kosterin 1999); Turkey (Kalkman et al. 2003; Miroglu and Kartal 2008; Salur and Mesci 2007; Salur and Ozsarac 2004); Iran (Ebrahimi et al. 2009; Ghahari et al. 2009); India (Subramanian 2009).
**Zoogeographic Affiliation:** Paleo-oriental.

***Ischnura forcipata* Morton, 1907**
**Own Records:** Loc. 21 - 3 ♂, 4 ♀; Loc. 29 4 ♂, 5 ♀; Loc. 33 - 1 ♂, 2 ♀; Loc. 34 - 4 ♂, 2 ♀; Loc. 19 - 3 ♂, 3 ♀; Loc. 2 - 6 ♂, 1 ♀; Loc. 12 - 3 ♂, 2 ♀; Loc. 83 - 5 ♂, 5 ♀; Loc. 86 - 4 ♂, 5 ♀; Loc. 85 - 5 ♂, 5 ♀; Loc. 84 - 4 ♀; Loc. 82 - 1 ♂, 2 ♀; Loc. 65 - 1 ♂, 4 ♀; Loc. 64 - 2 ♀; Loc. 59 - 2 ♂; Loc. 60 - 1 ♂, 3 ♀; Loc. 63 - 1 ♂, 2 ♀; Loc. 73 - 1 ♀; Loc. 72 - 2 ♀; Loc. 81 - 4 ♂, 1 ♀; Loc. 58 - 2 ♂, 2 ♀; Loc. 90 - 3 ♂, 5 ♀; Loc. 97 - 4 ♂, 2 ♀; Loc. 88 - 1 ♂; Loc. 92 - 2 ♂, 1 ♀; Loc. 167 1 ♂, 1 ♀; Loc. 169 - 1 ♀; Loc.168 - 2 ♂, 1 ♀; Loc. 170 - 3 ♂, 2 ♀; Loc. 171 - 1 ♂, 1 ♀; Loc. 165 - 1 ♂, 1 ♀; Loc. 162 - 2 ♂, 1 ♀; Loc. 161 - 3 ♀; Loc. 164 - 5 ♂, 2 ♀; Loc. 160 - 4 ♂, 2 ♀; Loc. 159 - 2 ♂, 2 ♀; Loc. 157 - 1 ♂; Loc. 156 - 2 ♂, 1 ♀; Loc. 153 - 3 ♀; Loc. 155 - 2 ♂, 1 ♀; Loc. 151 - 4 ♂, 2 ♀; Loc. 148 - 1 ♂; Loc. 149 - 1 ♂; Loc. 146 - 2 ♂, 5 ♀; Loc. 138 - 2 ♂; Loc. 140 - 1 ♂; Loc. 139 - 2 ♂, 1 ♀; Loc. 141 - 3 ♂, 2 ♀; Loc. 137 - 1 ♂, 4 ♀; Loc. 125 - 2 ♂, 2 ♀; Loc. 126 - 1 ♂; Loc. 113 - 1 ♂; Loc. 112 - 1 ♀; Loc. 116 - 1 ♂; Loc. 114 - 2 ♂; Loc. 115 - 1 ♂; Loc. 142 - 1 ♂; Loc. 144 - 3 S♂ 1 ♀; Loc. 118 - 1 ♀; Loc. 119 - 4 ♂; Loc. 120 - 3 ♂, 1 ♀.
**Previous Records from Pakistan:** This species was first reported from Pakistan (Baluchistan) by Fraser (1933). Kanth (1985) and Khaliq et al. (1990) reported it from Azad Jammu and Kashmir. Khaliq (1990) recorded its specimens from the Punjab, Baluchistan, and N.W.F.P. provinces. Khaliq and Maula (1999) also collected it from N.W.F.P. Again Khaliq and Siddique (1995), Zia et al. (2008), and Rafi et al. (2009) reported it from Azad Jammu and Kashmir. Zia et al (2009) collected specimens from northern Pakistan. Mitra and Babu (2009) reported it from the Punjab and Sindh provinces.
**Notes:** The areas of collection were rice fields and grasses grown among stagnant water and along running water bodies. Sometimes specimens were found flying among small grasses present a little distant to water streams.
**World Distribution:** Northern India and Baluchistan (Fraser 1933); Bhutan (Mitra 2006); India (Subramanian 2009); Iran (Ghahari et al. 2009).
**Zoogeographic Affiliation:** Paleo-oriental.

***Ischnura fountainei* Morton, 1905**
**Own Records:** Loc. 99 - 1 ♂, 5 ♀; Loc. 104 - 3 ♀.
**Previous Records from Pakistan:** Khaliq (1990) reported this species from Baluchistan province.
**Notes:** This species was collected from the margins of a water lake with not much vegetation around it, they were also recorded from the grassy margins of a running water stream.
**World Distribution:** Egypt (Geene 1994); Turkey (Dumont 1977; Kalkman et al. 2003).
**Zoogeographic Affiliation:** Afro-tropical and Palearctic.

***Ischnura inarmata* Calvert, 1898**
**Own Records:** Nil.
**Previous Records from Pakistan:** Mitra and Babu (2009) reported this species from Sindh and Punjab provinces.
**World Distribution:** India (Subramanian 2009)
**Zoogeographic Affiliation:** Oriental.

***Ischnura senegalensis* Rambur, 1842**
**Own Records:** Loc. 139 - 2 ♂; Loc. 141 - 3 ♂, 2 ♀; Loc. 123 - 1 ♂, 1 ♀; Loc. 126 - 1 ♀; Loc. 143 - 1 ♀.
**Previous Records from Pakistan:** Khaliq (1990) reported this species from Punjab, Sindh, and Baluchistan provinces. Khaliq and Siddique (1995), Zia et al. (2008), and Rafi et al. (2009) reported it from Azad Jammu and Kashmir. Zia et al. (2009) collected specimens from northern Pakistan. Mitra and Babu (2009) reported it from Punjab province.
**Notes:** This species was collected from different rice fields, slow-moving water bodies, and margins of a water lake and its out-flow.
**World Distribution:** India, Burma, Srilanka (Ceylon), Japan, Philippines, greater part of the African continent (Fraser 1933); Sudan (Dumont and Martens, 1984); Egypt (Geene 1994); Malawi (Barlow 1996); Hong Kong (Wilson 1997); Oman (Giles 1998); Africa, Middle East, India, Hong Kong, China (South and South East), and Seychelles (Ades and Kendrick 2004); Namibia, Sub-Saharan Africa, Asia from Levant to Japan and Philippines, and western Indian Ocean islands (Martens et al. 2003); India (Subramanian 2005; 2009); UAE (Feulner et al. 2007); Singapore (Rashid et al. 2008); China (Wilson et al. 2008); Expected in Turkey (Dumont 1977; Kalkman et al. 2003); Srilanka (Bedjanic and Conniff pers. comm.)
**Zoogeographic Affiliation:** Afrotropical and Paleo-oriental.

***Mortonagrion gautama* Fraser, 1923**
**Own Records:** Loc. 154 - 5 ♂.
**Previous Records from Pakistan:** The species was earlier reported by Hussain (2006) and Zia et al. (2009) from northern Pakistan.
**Notes:** This species was collected from stagnant water spots with very little vegetation around them.
**World Distribution:** India (Fraser 1933).
**Zoogeographic Affiliation:** Paleo-oriental.

***Pseudagrion ceylanicum* Kirby, 1891**
**Own Records:** Loc. 112 - 1 ♂.
**Previous Records from Pakistan:** Kanth (1985) collected this species from Azad Jammu and Kashmir.
**Notes:** A single specimen was collected from the bank of a big water reservoir.
**World Distribution:** Srilanka/Ceylon (Fraser 1933).
**Zoogeographic Affiliation:** Oriental.

***Pseudagrion decorum* Rambur, 1842**
**Own Records:** Loc. 39 - 3 ♂, 1 ♀; Loc. 42 4 ♂; Loc. 21 - 4 ♂, 4 ♀; Loc. 40 - 2 ♂; Loc. 52 - 2 ♀; Loc. 87 - 2 ♂, 1 ♀; Loc. 88 - 2 ♂, 3 ♀; Loc. 109 - 3 ♂, 1 ♀; Loc. 128 - 1 ♂, 1 ♀; Loc. 127 - 1 ♂, 2 ♀; Loc. 140 - 3 ♀; Loc. 113 - 1 ♂; Loc. 117 - 1 ♀; Loc. 119 - 1 ♂; Loc. 142 - 1 ♂.
**Previous Records from Pakistan:** Kanth (1985) and Khaliq et al. (1990) reported the species from Azad Jammu and Kashmir. Khaliq (1990) reported its presence in all four provinces. Khaliq and Siddique (1995), Zia et al. (2008), and Rafi et al. (2009) again reported this species from Azad Jammu and Kashmir. Recently Mitra and Babu (2009) reported it from Sindh province.
**Notes:** Specimens were recorded from rice fields and vegetation grown around water spots.
**World Distribution:** Throughout Continental India and Burma (Fraser 1933); Oman (Giles 1998); India (Subramanian 2009); Iran (Ghahari et al. 2009); UAE (Feulner et al. 2007); Srilanka (Bedjanic and Conniff pers. comm.).
**Zoogeographic Affiliation:** Paleo-oriental.

**Pseudagrion hypermelas Selys, 1876**
**Own Records:** Loc. 2 - 1 ♂, 1 ♀; Loc. 7 - 2 ♂, 2 ♀; Loc. 20 - 1 ♀; Loc. 28 - 1 ♂, 1 ♀; Loc. 21 - 1 ♂, 2 ♀; Loc. 34 - 2 ♂, 3 ♀; Loc. 33 - 1 ♀; Loc. 36 - 1 ♂, 3 ♀; Loc. 32 - 1 ♂, 1 ♀; Loc. 30 - 2 ♂, 2 ♀; Loc. 132 - 2 ♀; Loc. 126 - 2 ♂, 1 ♀; Loc. 128 - 1 ♂, 1 ♀; Loc. 123 -3 ♂, 2 ♀.
**Previous Records from Pakistan:** Kanth (1985) and Khaliq et al. (1990) reported this species from Azad Jammu and Kashmir. Khaliq (1990) collected specimens from the Punjab province. Recently Zia et al. (2008) and Rafi et al. (2009) reported this species from Azad Jammu and Kashmir. Mitra and Babu (2009) reported it from Punjab province.
**Notes:** Specimens were recorded from densely vegetated banks of water streams.
**World Distribution:** India (Fraser 1933; Subramanian 2009).
**Zoogeographic Affiliation:** Oriental.

***Pseudagrion laidlawi* Fraser, 1922**
**Own Records:** Loc. 44 - 3 ♂, 4 ♀; Loc. 41 2 ♀; Loc. 40 - 3 ♂, 2 ♀; Loc. 42 - 1 ♂, 3 ♀; Loc. 10 - 3 ♂, 1 ♀; Loc. 1 - 1 ♂, 1 ♀; Loc. 50 - 2 ♀; Loc. 89 - 1 ♂, 3 ♀; Loc. 87 - 1 ♂; Loc. 88 - 2 ♂, 2 ♀; Loc. 127 - 3 ♂, 2 ♀; Loc. 140 - 1 ♂, 1 ♀; Loc. 139 - 2 ♀; Loc. 141 - 2 ♂, 2 ♀.
**Previous Records from Pakistan:** This species was reported for the first time from Pakistan (Sindh, Karachi) by Fraser (1933). Kanth (1985) and Khaliq et al. (1990) reported this species from Azad Jammu and Kashmir. Khaliq (1990) recorded its presence in Punjab, Sindh, and N.W.F.P. provinces. Again Khaliq and Siddique (1995), Zia et al. (2008), and Rafi et al. (2009) reported it from Azad Jammu and Kashmir. Mitra and Babu (2009) reported it from Sindh and Punjab provinces.
**Notes:** Collections of specimens were made from rice fields, grassy margins of streams, rivers, and water lakes.
**World Distribution:** Pakistan, Sindh (Fraser 1933); India (Subramanian 2009).
**Zoogeographic Affiliation:** Oriental.

***Pseudagrion microcephalum* Rambur,**
**Own Records:** Nil.
**Previous Records from Pakistan:** Specimens were reported from the Sindh province by Mitra and Babu (2009).
**World Distribution:** Hong Kong, India, Srilanka, Nepal, Bangladesh, China, Japan, South East Asia, and Australia (Ades and Kendrick 2004); India (Subramanian 2005, 2009).
**Zoogeographic Affiliation:** Oriental and Australian.

***Pseudagrion rubriceps* Selys, 1876**
**Own Records:** Loc. 2 - 2 ♂, 1 ♀; Loc. 7 - 1 ♂, 1 ♀; Loc. 42 - 2 ♂, 3 ♀; Loc. 20 - 5 ♂, 4 ♀; Loc. 67 - 2 ♀; Loc. 76 - 1 ♂, 3 ♀; Loc. 57 - 1 ♂, 3 ♀; Loc. 51 - 1 ♂, 2 ♀; Loc. 56 - 2 ♂; Loc. 126 - 2 ♂; Loc. 128 - 1 ♂, 3 ♀; Loc. 127 - 1 ♀; Loc. 125 - 1 ♂; Loc. 112 - 1 ♂, 1 ♀; Loc. 140 - 1 ♂; Loc. 139 - 2 ♂, 1 ♀; Loc. 141 - 3 ♂, 2 ♀.
**Previous Records from Pakistan:** Kanth (1985) and Khaliq et al. (1990) reported this species from Azad Jammu and Kashmir. Khaliq (1990) reported it from the Punjab and N.W.F.P. provinces. Khaliq and Yousuf (1993a), Zia et al. (2008), and Rafi et al. (2009) again collected this species from Azad Jammu and Kashmir.
**Notes:** This species was found while resting over vegetation along a large reservoir of stagnant water. Specimens were also collected from rice fields and from vegetation present along running water. This species was mostly found in thin and short grassy vegetation in contrast to open lit, tall, and strong grasses at the same spots.
**World Distribution:** Plains and submountainous areas of Burma and Continental India, Malaysia, Indonesia (Java), Formosa, and Indo-China (Fraser 1933); Hong Kong (Wilson 1997); India (Subramanian 2005, 2009); China (Wilson et al. 2008); Singapore (Rashid et al. 2008). Ades and Kendrick (2004) documented this species' range including India, Nepal, Bangladesh, Hong Kong, South China, South East Asia, and Pakistan; Srilanka (Bedjanic and Conniff pers. comm.).
**Zoogeographic Affiliation:** Oriental.

***Pseudagrion spencei* Fraser, 1922**
**Own Records:** Loc. 39 - 1 ♂, 1 ♀; Loc. 10 2 ♂; Loc. 1 - 3 ♂ 1 ♀; Loc. 42 - 1 ♂, 1 ♀; Loc. 57 - 1 ♂, 1 ♀; Loc. 51 - 2 ♂, 1 ♀; Loc. 128 - 1 ♂; Loc. 112 - 1 ♂, 2 ♀; Loc. 101 - 1 S; Loc. 105 - 2 ♂, 1 ♀; Loc. 141 - 3 ♂, 2 ♀.
**Previous Records from Pakistan:** Kanth (1985) and Khaliq et al. (1990) reported this species from Azad Jammu and Kashmir. Khaliq (1990) reported it from Punjab, N.W.F.P., and Baluchistan provinces. Zia et al. (2008) and Rafi et al. (2009) again collected it from Azad Jammu and Kashmir.
**Notes:** Specimens were collected while preying at the bank of a water lake. Also, males were found flying over the surface of streams, and females were mostly recorded within and near vegetation.
**World Distribution:** India (Fraser 1933); Hong Kong (Wilson 1997); India, Pakistan, Nepal, Bangladesh, Hong Kong, and South China (Ades and Kendrick 2004).
**Zoogeographic Affiliation:** Oriental.

***Rhodischnura nursei* Morton, 1907**
**Own Records:** Loc. 28 - 2 ♂; Loc. 34 - 1 ♀; Loc. 19 - 1 ♂, 1 ♀; Loc. 67 - 1 ♂; Loc. 97 1 ♀; Loc. 127 - 1 ♀; Loc. 125 - 1 ♂; Loc. 123 - 1 ♂; Loc. 112 - 1 ♂.
**Previous Records from Pakistan:** Kanth (1985) collected this species from Azad Jammu and Kashmir. Khaliq (1990) documented its presence in all four provinces. Khaliq and Maula (1999) also recorded specimens from N.W.F.P. Khan et al. (2008), Zia et al. (2008), and Rafi et al. (2009) again collected this species from Azad Jammu and Kashmir. Mitra and Babu (2009) reported it from the Sindh and Punjab provinces.
**Notes:** Only a few specimens were found from each locality. Specimens were collected from grasses growing near standing and very slow-moving water bodies. They were also recorded from rice fields and the desert area.
**World Distribution:** India (Fraser 1933; Subramanian 2009).
**Zoogeographic Affiliation:** Paleo-oriental.

**Family Euphaeidae Selys, 1853 *Baydera indica* Selys, 1853**
**Own Records:** Loc. 5 - 1 ♂; Loc. 8 - 1 ♀; Loc. 76 - 3 ♀; Loc. 135 - 1 ♂; Loc. 131 - 1 ♀; Loc. 130 - 2 ♂, 1 ♀; Loc. 132 - 1 ♂; Loc. 126 - 1 ♀; Loc. 128 - 1 ♂, 2 ♀; Loc. 127 - 2 ♀; Loc. 142 - 1 ♂; Loc. 119 - 1 ♂; Loc. 113 - 1 ♂.
**Previous Records from Pakistan:** Kanth (1985) reported this species from Azad Jammu and Kashmir. Khaliq (1990) and Khaliq and Maula (1999) reported it from N.W.F.P. Again Khaliq et al. (1995), Zia et al. (2008), and Rafi et al. (2009) reported this species from Azad Jammu and Kashmir.
**Notes:** Specimens were collected while sitting over big stones and vegetation within slowmoving streams. Specimens were also found resting on rocks near water streams.
**World Distribution:** India (Fraser 1934; Subramanian 2009).
**Zoogeographic Affiliation:** Oriental.

***Bayadera longicauda* Fraser, 1928**
**Own Records:** Loc. 5 - 1 ♂; Loc. 8 - 1 ♀; Loc.13 - 2 ♂, 1 ♀; Loc. 135 - 4 ♀; Loc. 131 - 1 ♂; Loc. 126 - 1 ♀.
**Previous Records from Pakistan:** Specimens were earlier reported by Yousuf et al. (2000) and Zia et al. (2008) from Azad Jammu and Kashmir.
**Notes:** Specimens were found among tall and high vegetation beside water bodies.
**World Distribution:** India (Fraser 1934; Subramanian 2009).
**Zoogeographic Affiliation:** Oriental.

**Family Lestidae Calvert, 1901 *Lestes patricia* Fraser, 1924**
**Own Records:** Loc. 129 - 3 ♀.
**Previous Records from Pakistan:** This species was earlier reported from Azad Jammu and Kashmir by Rafi et al. (2009).
**Notes:** Specimens were recorded from bushy margins of a fast flowing stream.
**World Distribution:** India (Fraser 1933; Subramanian 2005; 2009).
**Zoogeographic Affiliation:** Oriental.

***Lestes praemorsus praemorsus* Selys, 1862**
**Own Records:** Loc. 129 - 2 ♂; Loc. 133 - 3 ♂ 1 ♀.
**Previous Records from Pakistan:** This species was reported for the first time from Pakistan by Luqman (unpubl). It was collected by Yousuf et al. (2000a) from Azad Jammu and Kashmir.
**Notes:** Specimens were recorded from a water spot with thin grasses around it.
**World Distribution:** India, Burma, and Philippines (Fraser 1933); Hong Kong, India, Srilanka, Nepal, southern China, and southeastern China (Ades and Kendrick 2004); India (Subramanian 2005, 2009).
**Zoogeographic Affiliation:** Oriental.

***Lestes thoracicus* Laidlaw, 1920**
**Own Records:** Loc. 128 - 3 ♂, 1 ♀; Loc. 127 - 2 ♀; Loc. 113 - 1 ♂; Loc. 117 - 1 ♂, 1 ♀; Loc. 118 - 3 ♀; Loc. 142 - 1 ♂.
**Previous Records from Pakistan:** Kanth (1985) and Khaliq et al. (1990) reported it from Azad Jammu and Kashmir. Khaliq (1990) collected specimens from the Sindh and Punjab provinces. Khaliq et al. (1995), Zia et al. (2008), and Rafi et al. (2009) collected this species again from Azad Jammu and Kashmir.
**Notes:** Specimens were caught over wild weeds and grasses near stagnant water spots.
**World Distribution:** India (Fraser 1933; Subramanian 2009).
**Zoogeographic Affiliation:** Oriental.

***Lestes umbrinus* Selys, 1891**
**Own Records:** Loc. 96 - 3 ♀, 2 ♂; Loc. 94 1 ♀; Loc. 91 - 1 ♂; Loc. 92 - 1 ♀; Loc. 134 1 ♂; Loc. 127 - 2 ♀; Loc. 117 - 2 ♀; Loc. 118 - 1 ♀.
**Previous Records from Pakistan:** Khaliq (1990) documented this species from the Sindh and Baluchistan province. Khan et al. (2008), Zia et al. (2008), and Rafi et al. (2009) reported this species from Azad Jammu and Kashmir.
**Notes:** Specimens were recorded from grasses and other dwarf vegetation near water streams in the Punjab and Baluchistan provinces. However, few females were collected from the spiny bushes in Umer Kot desert at Sindh during early hours of morning (06:00). It was noted that when the species was collected from the deserts of Mithi and Umer Kot, there was no water available for miles. However, when collected from other spots there was running as well as standing water spots in close proximity. Specimens were also recorded while feeding among grasses and other vegetation near some water streams.
**World Distribution:** Yunnan, India, and Burma (Fraser 1933); India (Subramanian 2009).
**Zoogeographic Affiliation:** Paleo-oriental.

***Lestes viridulus* Rambur, 1842**
**Own Records:** Loc. 10 - 1 ♀; Loc. 139 - 1 ♂; Loc. 137 - 1 ♂, 1 ♀.
**Previous Records from Pakistan:** This species was reported earlier by Khaliq (1990) from the Punjab province. Khaliq et al. (1995), Zia et al. (2008), and Rafi et al. (2009) reported it from Azad Jammu and Kashmir.
**Notes:** Specimens were caught on the banks of streams with grassy vegetations around them.
**World Distribution:** India (Fraser 1933; Subramanian 2009).
**Zoogeographic Affiliation:** Oriental.

***Cylonolestes cyanea* Selys, 1930**
**Own Records:** Loc. 122 - 2 ♂; Loc. 128 - 1 ♂.
**Previous Records from Pakistan:** This species was reported for the first time from Pakistan by Luqman (unpubl). It was collected by Yousuf et al. (2000a) from Azad Jammu and Kashmir.
**Notes:** Specimens were recorded from dense, bushy vegetation along fast-moving water streams as well as from the bank of a standing water lake.
**World Distribution:** India and northern Punjab (Fraser 1933).
**Zoogeographic Affiliation:** Oriental.

**Family Platycnemidae Tillyard, 1917 *Calicnemis eximia* Selys, 1863**
**Own Records:** Loc. 7 - 2 ♂, 1 ♀; Loc. 5 - 2 ♂, 2 ♀; Loc. 8 - 2 ♂, 2 ♀; Loc. 61 - 1 ♂; Loc. 62 - 1 ♂, 1 ♀; Loc. 63 - 1 ♂; Loc. 59 2 ♀; Loc. 58 - 6 ♂, 5 ♀; Loc. 81 - 3 ♂, 4 ♀; Loc. 79 - 2 ♂, 2 ♀; Loc. 80 - 5 ♂, 2 ♀; Loc. 65 - 1 ♂; Loc. 66 - 6 ♂, 8 ♀; Loc. 76 - 3 ♀; Loc. 130 - 6 ♂, 5 ♀; Loc. 135 - 1 ♂, 2 ♀; Loc. 136 - 1 ♂, 1 ♀; Loc. 133 - 4 ♂, 2 ♀; Loc. 131 - 7 ♂, 5 ♀; Loc. 129 - 3 ♂, 1 ♀; Loc. 132 - 1 ♀; Loc. 126 - 2 ♂; Loc. 128 - 2 ♀.
**Previous Records from Pakistan:** Khaliq (1990) documented this species from the Punjab and N.W.F.P. provinces. Khaliq and Maula (1999) also collected specimens from N.W.F.P. Khaliq et al. (1990), Zia et al. (2008), and Rafi et al. (2009) reported this species from Azad Jammu and Kashmir.
**Notes:** Specimens were collected from spots with water flowing very slowly, from swampy places, rice fields, and dwarf grasses near water bodies. A few specimens were collected from vegetation on small mountains near water streams. This species is never observed with much close human disturbance.
**World Distribution:** India (Fraser 1933; Subramanian 2009); Bhutan (Mitra 2006).
**Zoogeographic Affiliation:** Oriental.

***Coeliccia renifera* Selys, 1886**
**Own Records:** Loc. 4 - 1 ♂; Loc. 15 - 2 ♂, 1 ♀; Loc. 135 - 3 ♂; Loc. 140 - 1 ♂.
**Previous Records from Pakistan:** Khaliq (1990) collected this species from the Punjab province. Zia et al. (2008) reported it from Azad Jammu and Kashmir.
**Notes:** Specimens were recorded from grassy and weedy spots. A few spots were very damp and specimens were present under the dense shade of trees; no sunlight could reach these spots. At such spots, they were observed to rest on stones repeatedly after short flights.
**World Distribution:** India (Fraser 1933; Subramanian 2009); Bangladesh and Nepal (Shigeru 2000); China (Wilson et al. 2008).
**Zoogeographic Affiliation:** Oriental.

***Copera ciliata* Selys, 1863**
**Own Records:** Loc. 39 - 1 ♂, 1 ♀.
**Previous Records from Pakistan:** Khaliq (1990) caught this species from the Punjab province.
**Notes:** The specimens were found hiding and feeding within dense vegetation along the margins of a large, weedy pond present near Chenab River.
**World Distribution:** Hong Kong (Wilson 1997); Bangladesh, China, and South East Asia (Ades and Kendrick 2004); India (Subramanian 2009); China (Wilson et al. 2008; Choong et al. 2008).
**Zoogeographic Affiliation:** Oriental.

***Copera marginipes* Rambur, 1842**
**Own Records:** Loc. 10 - 5 ♂, 3 ♀; Loc. 20 2 ♂, 3 ♀; Loc. 56 - 5 ♂, 2 ♀; Loc. 50 - 1 ♂; Loc. 58 - 1 ♀; Loc. 57 - 2 ♂, 1 ♀; Loc. 126 1 ♂; Loc. 127 - 1 ♂, 1 ♀; Loc. 112 - 1 ♀.
**Previous Records from Pakistan:** Kanth (1985) reported this species from Azad Jammu and Kashmir. Khaliq (1990) reported it from the Punjab and N.W.F.P. provinces. Khan et al. (2008), Zia et al. (2008), and Rafi et al. (2009) collected this species from Azad Jammu and Kashmir. Recently Mitra and Babu (2009) again reported specimens from the Punjab province.
**Notes:** Specimens were caught from the margins of different stagnant water spots and running water streams.
**World Distribution:** Southern Asia, India, Srilanka (Ceylon), Siam, Burma, and Sondaic Islands (Fraser 1933); Hong Kong (Wilson 1997); Bangladesh, India, Hong Kong, China, Srilanka, Nepal, and south-eastern China (Ades and Kendrick 2004); India (Subramanian 2005; 2009); China (Wilson et al. 2008; Choong et al. 2008); Singapore (Rashid et al. 2008); Srilanka (Bedjanic and Conniff pers. comm.).
**Zoogeographic Affiliation:** Paleo-oriental.

***Platycnemis dealbata* Selys, 1850**
**Own Records:** Nil.
**Previous Records from Pakistan:** Mitra and Babu (2009) reported this species from the Punjab province.
**World Distribution:** Iran (Ghahari et al. 2009; Kalkman et al. 2003); India (Subramanian 2009).
**Zoogeographic Affiliation:** Paleo-oriental.

**Family Platystictidae Laidlaw, 1924 *Drepanosticta polychromatica* Fraser, 1931**
**Own Records:** Loc. 10 - 1 ♂; Loc. 3 - 1 ♂.
**Previous Records from Pakistan:** Khaliq (1990) reported this species from the Punjab province.
**Notes:** Specimens were collected over a water lake, a bank of running water spot, and from a spring. All spots were surrounded by dense grasses and wild flora.
**World Distribution:** India (Fraser 1933; Subramanian 2009).
**Zoogeographic Affiliation:** Oriental.

**Family Protoneuridae Tillyard, 1917 *Elattoneura atkinsoni* Selys, 1886**
**Own Records:** Loc. 2 - 2 ♂, 2 ♀.
**Previous Records from Pakistan:** This species was recorded for the first time from Pakistan.
**Notes:** Specimens were collected from outflow of surplus water from Simly Dam. One specimen was collected on a wing from vegetation surrounding flowing water; however, two specimens were collected by blind sweeping in a cave under a big rock stone. The cave was just a few feet away from water.
**World Distribution:** India (Fraser 1933; Subramanian 2009).
**Zoogeographic Affiliation:** Oriental.

***Elattoneura nigerrima* Laidlaw, 1935**
**Own Records:** Loc. 126 - 1 ♂, 2 ♀; Loc. 128 - 2 ♀.
**Previous Records from Pakistan:** Kanth (1985) and Khaliq et al. (1990) reported this species from Azad Jammu and Kashmir. Khaliq (1990) reported specimens from the Punjab and N.W.F.P. provinces. Zia et al. (2008) and Rafi et al. (2009) collected this species again from Azad Jammu and Kashmir.
**Notes:** A few specimens were collected while ravening near a lake. Also some specimens were recorded over grassy vegetation grown around water bodies.
**World Distribution:** India (Fraser 1933; Subramanian 2009).
**Zoogeographic Affiliation:** Oriental.

***Elattoneura souteri* Fraser, 1924**
**Own Records:** Loc. 10 - 4 ♂, 4 ♀; Loc. 11 2 ♂; Loc. 64 - 8 ♂, 5 ♀; Loc. 117 - 1♂ 1♀.
**Previous Records from Pakistan:** This species was recorded for the first time from Pakistan.
**Notes:** The species was recorded from variable habitats, i.e. from very slow moving water near the ground, fast running water of streams, marshes, bogs and bushes, and climbers and creepers near water. This species was collected in open sunlit areas as well as from moist and humid stones present under dense shady trees.
**World Distribution:** India (Fraser 1933; Subramanian 2009).
**Zoogeographic Affiliation:** Oriental.

***Elattoneura tetrica* Laidlaw, 1917**
**Own Records:** Nil.
**Previous Records from Pakistan:** Mitra and Babu (2009) reported this species from the Punjab province.
**World Distribution:** India (Subramanian 2005; 2009).
**Zoogeographic Affiliation:** Oriental

**Family Synlestidae *Megalestes major* Selys, 1962**
**Own Records:** Loc. 2 - 3 ♂, 3 ♀; Loc. 5 - 4 ♂, 2 ♀; Loc. 4 - 2 ♂, 5 ♀; Loc. 8 - 3 ♂, 1 ♀; Loc. 15 - 5 ♂, 6 ♀; Loc. 58 - 3 ♂, 2 ♀; Loc. 66 - 2 ♂; Loc. 75 - 1 ♂, 3 ♀; Loc. 74 - 2 ♂, 1 ♀; Loc. 130 - 2 ♂, 1 ♀; Loc. 133 - 1 ♂, 1 ♀; Loc. 134 - 1 ♂, 3 ♀; Loc. 135 - 5 ♂; Loc. 126 - 4 ♂, 4 ♀; Loc. 128 - 1 ♂, 2 ♀; Loc. 118 - 1 ♀.
**Previous Records from Pakistan:** Kanth (1985) reported this species from Azad Jammu and Kashmir. Khaliq (1990) collected specimens from the Punjab and N.W.F.P. provinces. Khaliq and Maula (1999) also documented its presence in N.W.F.P. Khaliq et al. (1995), Khan et al. (2008), Zia et al. (2008), and Rafi et al. (2009) again collected specimens from Azad Jammu and Kashmir. Zia et al (2009) reported specimens from northern Pakistan.
**Notes:** Specimens were found sitting over tall, grassy vegetation beside water bodies as well as on nearby small mountains. Some specimens were collected from rice fields and swampy spots along the banks of fast running streams. At dusk, they were observed to hide under mountain cover.
**World Distribution:** India, Nepal, northern Punjab in Pakistan (Fraser 1933); Bhutan (Mitra 2006); India (Subramanian 2009).
**Zoogeographic Affiliation:** Paleo-oriental.

## Discussion

The distribution of zygoptera during this study shows very interesting results and confirms zygopterous fauna in all three biogeographic regions, i.e. Palearctic, Oriental, and Afrotropical. Zoogeographic affiliation for each species is provided on the basis of major works on biogeographic regions of Pakistan, including Atlas of Pakistan (1997) and Rafi et al. (2010). Results indicate that the damselfly fauna of Pakistan includes 53 taxa within 21 genera (Table 2).

The biogeographic distribution of *N. chinensis* of the family Calopterygidae shows its wide— spread status throughout northern hilly tracts. This species is also reported from India, which substantiates its oriental distribution; however, it is still not known from Iran and Afghanistan.

Family Chlorocyphidae represents seven taxa under two genra, i.e. *Libellago and Rhinocypha.* Genus *Libellago* is reported with two taxa, i.e. *L. greeni and L. lineata lineata.* Among these, *L. greeni* showed limited distribution, and is only recorded from northern areas whose extremes come under the Palearctic portion of the country with boundaries along China and Afghanistan. Its world distribution shows its occurrence in Sri Lanka, an Oriental country (Bedjanic and Conniff pers. comm.). It can therefore be considered as a Paleo-oriental species. Further work is suggested, however, for knowing its exact Zoogeographic status. Another taxon of the genus is a subspecies, i.e. *L. lineata lineate,* which is a new record for Pakistan, and its area of collection is closely bordered with the Oriental areas of country. Its distribution, as reported by Silsby (2001) and Bedjanic and Conniff (Pers. comm), is from Sri Lanka, and Subramanian (2005) reports it from India; these reports stress its Oriental status. The second genus, Rhinocypha, has an Oriental origin which includes five taxon named *R. hilaryae, R. immaculata, R. quadrimaculata, R. trifasciata,* and *R. unimaculata.* With the exception of *R. hilaryae,* which has its distribution to upper Myanmar, Burma (Fraser 1933); all other four taxon are confined to subcontinent only (India and Pakistan).

**Table 2.**
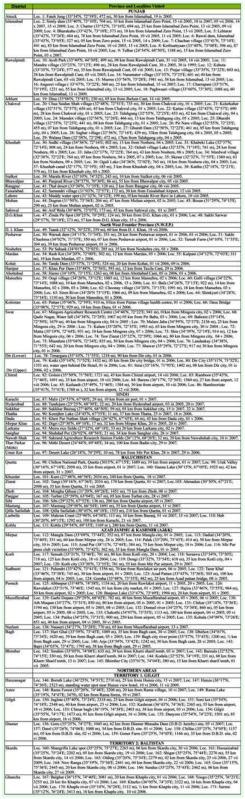
Localities visited for collecting Zygoptera of Pakistan during 2004–2009

The largest family for Zygoptera of Pakistan with 26 taxa is Coenagrionidae with eight genera. Most of the species in this family are affiliated with Oriental biogeography. Yet taxa including *Ischnura elegans, I. forcipata, Mortonagrion gautama, Pseudagrion decorum,* and *Rhodischnura nursei* have Palaeo-Oriental distribution. However, *I. fountainei* shows both Palearctic as well as Afro-tropical and *I. senegalensis* shows Afrotropical and Paleo-oriental distribution. Species like *Agriocnemis pygmaea* appear to be wide-spread, having affiliation with Paleooriental and Australian biogeographies. *Pseudagrion microcephalum* shows Oriental and Australian distribution. *Enallagma cyathigerum* has Palearctic as well as Nearctic distribution. However, *Ischnura aurora* is, again, a widely-distributed species and occurs in Australian, Paleo-oriental, and Afrotropical biogeographies.

The family Euphaeidae with two taxa, i.e. *Bayadera longicauda* and *B. indica,* shows Oriental distribution. Taxa under the family Lestidae, i.e. *Lestes patricia, L. p. praemorsus, L. thoracicus, L. viridulus,* and *Cylonolestes cyanea,* are also Oriental in distribution; while *L. umbrinus* is observed to be a Paleo-oriental species. Three taxa under the family Platycnemidae, i.e. *Calicnemis eximia, Coeliccia renifera,* and *Copera ciliate,* represent their Oriental status. However, *Copera marginipes* and *Platycnemis dealbata* of same family represent Paleo-oriental distribution. Families like Platystictidae with the taxon *Drepanosticta polychromatica;* and Protoneuridae with the taxa *Elattoneura atkinsoni, E. souteri, E. nigerrima,* and *E. tetrica;* have their Zoogeographic affiliation with Oriental region. The family Synlestidae has a single taxa, i.e. *Megalestes major,* with Paleo-oriental representation.

The above results indicate that Zygoptera fauna of Pakistan are mostly Oriental with 37 taxa, however, 9 taxa have Paleo-oriental distribution while rest are affiliated with mixed contribution of Afrotropical, Australian, Palearctic, Nearctic, Oriental, and Neotropic biogeographies. Keeping in mind the topography of the country, known fauna seems to be incomplete. The number is much less than Srilanka where a total of 52 damselflies species have been recorded (Bedjanic and Coniff pers. comm.). From India, 375 zygopterous species are reported (Subramanian 2009). A small country like Nepal has an odonate fauna of 180 species (Singh 1995). This indicates the need of further surveys and taxonomic research to develop the unexplored zygopterous fauna of Pakistan.

## Figures and Tables

**Appendix 1.**
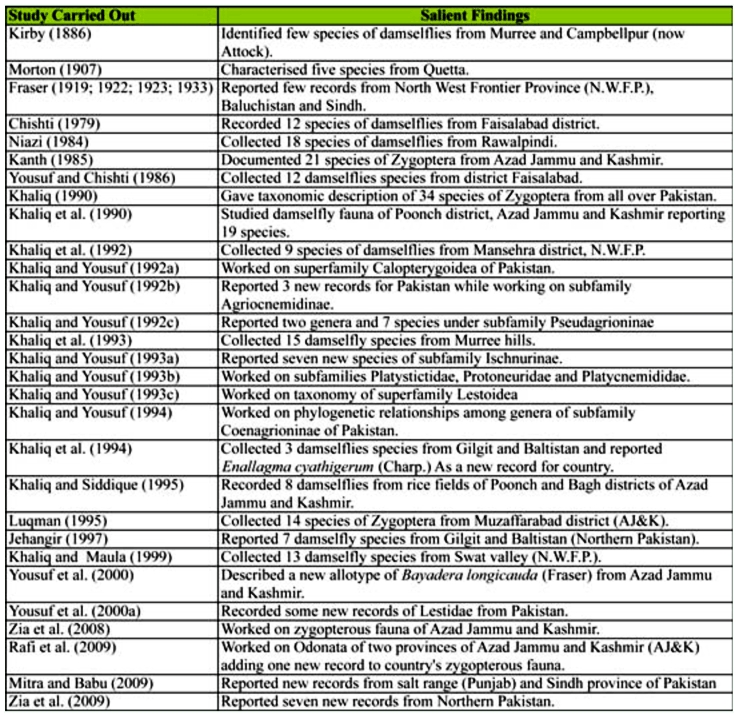
Overview of taxonomic work already done on Zygoptera of Pakistan.
